# Mobilisation communautaire pour l'amélioration de la surveillance de la grossesse en milieu rural au Maroc

**DOI:** 10.11604/pamj.2020.35.73.18328

**Published:** 2020-03-16

**Authors:** Majda Sebbani, Latifa Adarmouch, Mohamed Amine, Mohamed Cherkaoui

**Affiliations:** 1 Service de Recherche Clinique, CHU Mohammed VI de Marrakech, Marrakech, Maroc; 2 Département de Médecine Communautaire et de Santé Publique, Laboratoire PCIM, Faculté de Médecine et de Pharmacie de Marrakech (FMPM), Université Cadi Ayyad, Marrakech, Maroc; 3 Laboratoire d'Ecologie Humaine, Département de Biologie, Faculté de Sciences, Université Cadi Ayyad de Marrakech, Marrakech, Maroc

**Keywords:** Recherche action, participation communautaire, surveillance de grossesse, Maroc, Action research, community engagement, monitoring in pregnant women, Morocco

## Abstract

**Introduction:**

L'amélioration de la santé maternelle est une priorité de santé pour de nombreux pays en développement. L'objectif était de décrire l'apport d'un projet à base communautaire dans l'amélioration du suivi de la grossesse d'une commune rurale vulnérable du Haut Atlas marocain.

**Méthodes:**

Il s'agissait d'une recherche action intégrant une démarche de mobilisation communautaire menée en 2014. Le projet impliquant plusieurs intervenants dont les acteurs sociaux, les autorités locales, les professionnels de santé et des étudiants en médecine a permis d'assurer le suivi d'une cohorte de 283 femmes enceintes. Au cours des consultations prénatales les femmes étaient interviewées, examinées et participaient à des séances d'information et d'éducation. Les enquêtrices parlant berbères étaient formées pour la collecte des données. Les analyses statistiques étaient descriptives et bivariées avec un seuil de signification de 5%.

**Résultats:**

La moyenne de l'âge des participantes était de 27,1±6,7 ans. La majorité était analphabète, sans couverture sanitaire et sans profession. Près de 73,4% n'avaient aucun contact antérieur avec le système de soins (N = 252). Parmi 500 grossesses attendues estimées, 56,6% des femmes étaient suivies, 30,4% ont consulté deux fois et 6,7% ont effectué trois visites sur un total de 407 consultations prénatales effectuées. L'adhésion au suivi de la grossesse par la réalisation d'au moins deux consultations a été de 60,3%. La notion d'anomalie du déroulement de la grossesse était significativement liée au suivi (p = 0,04).

**Conclusion:**

Les résultats témoignent de l'intérêt de la participation communautaire dans l'approche des problèmes de santé complexes comme la santé maternelle.

## Abstract (en)

**Introduction:**

The improvement of maternal health is a health priority in many developing countries. The purpose of this study was to describe the contribution of a community project in the improvement of monitoring during pregnancy in a vulnerable rural commune in the Moroccan High Atlas.

**Methods:**

We conducted an action-research project incorporating an approach for community mobilization conducted in 2014. The project involving several participants such as social actors, local authorities, health professionals and students in medicine helped to ensure the monitoring of a cohort of 283 pregnant women. In the course of antenatal consultations, women were interviewed, assessed and participated in information sessions and education. The interviewers speaking berber were trained to data collection. Bivariate and descriptive statistical analyzes were carried out, with a threshold for significance of 5%.

**Results:**

The average age of patients was 27.1± 6.7 years. The majority of them was illiterate, without health coverage and with no career. Nearly 73.4% had no previous contact with the health care system (N = 252). Among 500 planned pregnancies, 56.6% of women were monitored, 30.4% consulted twice and 6.7% underwent three consultations out of a total of 407 antenatal consultations performed. Follow-up compliance by undergoing at least two consultations was 60.3%. Abnormality detection during pregnancy was significantly associated with monitoring (p = 0.04).

**Conclusion:**

The results reflect the importance of community engagement in the approach to complex health problems as maternal health.

## Introduction

La surveillance médicale de la grossesse par la réalisation des consultations prénatale (CPN) est l'un des quatre axes d'intervention fondamentales pour une maternité sans risque [1]. La CPN vise à dépister et à prévenir les complications maternelles et fœtales, et de les traiter au moment opportun. C'est aussi l'occasion pour les professionnels de soins d'approcher les femmes et leurs familles, de les informer sur les avantages de l'accouchement médicalisé et de les fidéliser aux structures de soins [2, 3]. Au Maroc, la consultation prénatale est organisée au niveau des structures de soins de santé primaires, des maisons d'accouchement, des maternités hospitalières et au niveau des points de contact des équipes mobiles. Conformément aux recommandations internationales, quatre visites prénatales sont recommandées pour toute grossesse normale. Cependant, ces recommandations ne sont pas toujours respectées pour plusieurs raisons économiques, sociales et culturelles. En effet, selon l'enquête nationale sur la population et la famille de 2011, 77,1% des femmes enquêtées ont bénéficié d'une consultation prénatale durant leur dernière grossesse (contre 67,8% en 2004). Cette proportion est nettement inférieure en milieu rural (62,7% en milieu rural versus 91,6% en milieu urbain) [4]. Cette disparité est expliquée par multiples déterminants qui caractérisent le milieu rural au Maroc. La vulnérabilité socioéconomique de la femme rurale s'ajoute aux barrières d'accès aux soins d'ordre structurel et culturel et entrave la démarche de surveillance des grossesses. Si l'on admet que l'amélioration des indicateurs d'utilisation des services de santé est en partie en relation avec la disponibilité et la qualité de ces services, le recours et l'utilisation des services relèvent de comportements individuels qui reflètent le degré de conscience de la population et son implication dans la gestion de la santé. L'élaboration des stratégies à l'échelle nationale doit prendre en compte ces variances intra-régionales. A cet effet, l'approche promotion de la santé intègre des stratégies complémentaires qui visent le problème selon différents angles de vue (du point de vue des décideurs, de la collectivité et de l'individu) qui permettent d'agir ensemble pour minimiser les iniquités d'accès aux soins persistantes entre le milieu urbain et rural. La recherche action, qui est l'approche privilégiée par les acteurs en promotion de la santé est une démarche à la fois productive de connaissances et capable d'apporter des modifications du réel en prenant en considération le facteur humain [5]. La création d'un partenariat entre les chercheurs, les acteurs, et la population pourrait être une alternative efficace pour la résolution de problème de santé. La faculté de médecine pourrait jouer un rôle crucial comme acteur engagé dans la concrétisation du concept de responsabilité sociale à travers des actions qui visent les réels besoins de santé de la population [6]. Des projets qui intègrent toutes ces dimensions sont encore peu fréquents dans notre contexte, et les résultats disponibles en matière de santé maternelle proviennent en général d'enquête ou d'étude menée par le ministère de la santé. L'objectif du travail était de décrire l'apport d'un projet à base communautaire porté par des étudiants en médecine dans l'amélioration du suivi de la grossesse par les femmes enceintes d'une commune rurale vulnérable du Haut Atlas occidental au Maroc.

## Méthodes

### Contexte

Il s’agissait d'une commune rurale montagneuse, située au flanc nord de la chaine du Haut Atlas occidental à 78 km de la ville de Marrakech. Sa position sur le Haut Atlas lui confère un relief très accidenté. En hiver surtout, les intempéries font que certains « douars » (ensemble d'habitats) sont parfois enclavés. Le taux de pauvreté est estimé à 23,9% en 2007 selon le Haut-commissariat au plan marocain [7]. Il est nettement supérieur à la moyenne nationale qui est de 8,9%; ce qui qualifie la commune parmi les plus pauvres de la région. Concernant la couverture sanitaire, elle est desservie par un seul centre de santé, et une maison d'accouchement localisée à 23 Km ainsi que trois dispensaires ruraux et un hôpital provincial localisé à 87 Km. Pour accéder au centre de santé de référence, quelques véhicules (de type transit) assurent le déplacement de la population, mais dans des conditions précaires. La recherche action visant la promotion de la santé maternelle au niveau de la commune comportait plusieurs volets complémentaires: le diagnostic de la situation à travers des enquêtes connaissances attitudes et pratiques menées auprès des hommes [8] et des femmes de la région, des entretiens et de l'observation du terrain; le suivi de la grossesse des femmes durant une année; l'éducation pour la santé des femmes sous forme de séances en groupe nommées « classes des mères »; la sensibilisation de la population par les acteurs communautaires, les associations locales et les étudiants en médecines. Le plaidoyer auprès des institutions sanitaires et des décideurs. L'étude présentée dans cet article s'inscrit dans le volet suivi de la grossesse.

### Méthodologie de l'étude

Il s'agissait d'une étude interventionnelle longitudinale qui a consisté à mettre en place un programme de surveillance des grossesses par une équipe mobile de professionnels de santé et d'étudiants en médecine volontaires suivre une cohorte de femmes enceintes au niveau de la commune, évaluer leur degré d'adhésion au programme proposé et étudier les facteurs associés à cette adhésion. La population cible était les femmes déclarées enceintes durant la période de l'étude (année 2014) et résidentes dans la commune ou sa région. L'échantillonnage était de type exhaustif incluant toutes les femmes enceintes qui se sont présentées pour le suivi de leur grossesse au niveau du centre de santé local. Un recensement des femmes enceintes au niveau de la commune par les autorités locales a précédé l'étude de deux mois. Les femmes ont été initiées au déroulement du projet et invitées à participer par les agents de la commune, les membres d'une association d'étudiants en médecine de la faculté de médecine et de pharmacie de Marrakech actifs au niveau de la zone d'étude et par les adhérents à un espace associatif local. Ainsi près de 500 grossesses ont été attendues durant la période du projet selon les estimations.

Les données collectées ont été regroupées selon les sections suivantes: caractéristiques sociodémographiques avec l'âge, le niveau de scolarisation, la couverture médicale et la consanguinité (lien de parenté de 1^er^ ou de 2^e^ degré avec le conjoint); les antécédents familiaux et personnels; l'utilisation antérieure aux soins maternels; le suivi de la grossesse actuelle et son terme (premier, second ou troisième trimestre). Un dossier « suivi de la femme » a été conçu en se basant sur le dossier médical utilisé au niveau du centre de santé ainsi que celui établi par le ministère de la Santé marocain. Les données ont été collectées à partir de l'interrogatoire de la femme assuré par des enquêteurs formés et de l'examen physique réalisé par le médecin gynécologue (une femme) et des étudiantes en médecine en 3^e^ cycle, en présence d'une sage-femme ou d'un médecin généraliste. La consultation se déroulait en quatre temps: (1) examen général avec prise du poids, taille, température, tension artérielle et bandelettes urinaires; (2) examen gynécologique avec hauteur utérine, activité cardiaque du fœtus, mouvements actifs fœtaux. Un examen au spéculum était obligatoire au moins une fois pour chaque femme associé à un toucher vaginal pour préciser l'état du bassin, du col et de la présentation fœtale; (3) échographie obstétricale; (4) prélèvement pour analyse biologique réalisé par un infirmier ou un étudiant en médecine. Les étudiants en médecine volontaires ont été formés avant le démarrage de l'étude. Le recrutement des enquêtrices parlant amazigh a été privilégié vu la spécificité culturelle de la région. La saisie et la gestion des données ont été assurées par des coordonnateurs du projet. Le logiciel SPSS version 16 a été utilisé lors de l'analyse statistique. Les variables qualitatives ont été décrites par les effectifs et les proportions, les variables quantitatives par leurs mesures de tendance centrale et de dispersion. L'analyse bi variée a consisté en la comparaison de deux proportions par le test de Fisher, et la comparaison de moyennes par le test t de Student avec un seuil de signification statistique de 5%.

Concernant la terminologie, le terme « CPN » a été utilisé pour signifier le trimestre de grossesse auquel la femme a été vue et examinée. CPN 1: consultation prénatale au 1^er^ trimestre; CPN2: consultation prénatale au 2^e^ trimestre; CPN3: consultation prénatale au 3^e^ trimestre. Le terme suivi de la grossesse a été adopté pour décrire la surveillance du déroulement de la grossesse par l'équipe mobile de médecins dans le cadre du projet. Les anomalies de déroulement de la grossesse actuelle renvoyaient à la notion de complications ou de comorbidités associées découverte au cours de la grossesse (HTA, diabète, infection, saignement, anémie). Pour les grossesses antérieures, c'était l'antécédent d'une fausse couche, d'avortement, de mort fœtal in utéro ou d'autres complications. Cette étude a été conduite après l'obtention de toutes les autorisations administratives nécessaires. Les aspects éthiques ont été respectés, en occurrence les bénéfices escomptés, l'anonymat, la confidentialité et le respect de la personne. Le consentement oral des femmes a été obtenu avant leur inclusion.

## Résultats

Au Total, 283 femmes enceintes ont participé à l'étude et ont bénéficié d'un suivi de leur grossesse dans le cadre du projet.

**Les caractéristiques sociodémographiques des participantes:** la moyenne de l'âge était de 27,1±6,7 ans. Parmi les répondantes, la majorité était analphabète, sans couverture sanitaire et sans profession. La notion de consanguinité avec le conjoint était notée chez 32,5% des femmes (Tableau 1).

**Tableau 1:** Les caractéristiques des participantes

Les caractéristiques des femmes	Effectif	Pourcentage %
**Niveau d’instruction (N=269)**		
Aucun	179	66,6
Primaire	82	30,5
Secondaire	8	3,0
**Couverture sanitaire (N=254)**		
Oui	13	4,6
Non	241	85,2
**Consanguinité (N=271)**		
Oui	88	32,5
Non	183	67,5
**Primiparité**	39	13,8

**Les antécédents médicaux et obstétricaux:** la notion d'HTA (hypertension artérielle) familiale a été rapportée dans 23,7% des réponses, le diabète l'était dans 32,7% (N = 257). L'antécédent d'anomalies au cours d'une grossesse antérieure a été noté dans 22,4% des cas (N = 236) dominé par l'avortement et la mort fœtal in utéro. L'expérience d'une mortinatalité ou d'un décès néonatal précoce était rapportée par six femmes. Les causes n'étaient pas connues. Quant à la notion d'anomalie du déroulement de la grossesse actuelle, elle était rapportée par la moitié des femmes. L'infection et l'anémie étaient les plus citées (82,7 % et 17,2% respectivement) (Tableau 2).

**Tableau 2:** Les antécédents et morbidités auto-déclarées par les participantes

les antécédents et morbidités auto-déclarées par les participantes	Effectif	Pourcentage %
Les anomalies au cours de la grossesse déclarées par les femmes	156	55,1
L’infection (urinaire ou génitale)	129	82,7
L’anémie	27	17,3
L’HTA	07	4,5
Le saignement	07	4,5
Le diabète	02	1,3
Les anomalies de déroulement des grossesses antérieures (N=236)	63	26,7
Avortement	33	14,0
Mort fœtal in utéro	18	7,6
Accouchement prématuré	02	0,8

**L'utilisation des soins de santé maternelle:** en ce qui concerne l'utilisation des services de santé maternelle au cours des grossesses antérieures, 73,4% des femmes ont déclaré qu'elles n'avaient aucun contact avec le système de soins et qu'elles n'avaient pas suivi la grossesse (N = 252). L'expérience d'un accouchement dans une structure sanitaire ou assisté par un personnel qualifié était rapportée par 26% des femmes. Parmi les 500 grossesses attendues, 56,6% des femmes enceintes ont adhéré au projet. Au total 407 consultations prénatales ont été effectuées au cours de l'étude. La plupart était au cours du 3^e^ trimestre de grossesse (72,8%), suivi du 2^e^ trimestre (53%) et du premier dans la plus faible proportion (18%). Quant au suivi de la grossesse par les participantes, 30,4 % des femmes ont consulté deux fois au cours de l'étude et 6,7% ont effectué trois visites. Les résultats dépendaient du terme auquel la femme a été vue à la première consultation (Tableau 3).

**Tableau 3:** Utilisation des soins de santé maternelle par les femmes avant et au cours du projet

Utilisation des soins de santé maternelle	Effectif	Pourcentage %
**Utilisation antérieure des services de santé maternelle (N=252)**		
Non	185	73,4
Oui	67	26,6
**Le terme de consultation prénatale par la femme au cours du projet (CPN)**		
CPN1	22	7,8
CPN2	47	16,6
CPN3	109	38,5
CPN1 et CPN2	08	2,8
CPN1 et CPN3	02	0,7
CPN2 et CPN3	76	26,9
CPN1 et CPN2 et CPN3	19	6,7

Les facteurs associés à l'adhésion des femmes au suivi de leur grossesse. L'adhésion au suivi de la grossesse dans le cadre du projet par la réalisation d'au moins deux consultations (CPN) pour les femmes vues en 1^er^ ou 2^e^ trimestre (N = 174) a été de 60,3%. L'étude des facteurs qui pouvaient expliquer cette adhésion n'a pas montré de différence entre les deux groupes de femmes sauf pour la perception d'anomalie du déroulement de la grossesse qui était significativement liée au suivi avec un p = 0,04 (Tableau 4).

**Tableau 4:** Les facteurs associés à l’adhésion des femmes au suivi de leur grossesse dans le cadre du projet

Les facteurs	Adhésion		P
	Non (N=69)	Oui (N=105)	
	Effectif n (%)	Effectif n (%)
**Age de la femme en année (moyenne ±écart type)**	27,6 ±7,6	26,9 ± 6,1	0,54
**Instruction de la femme (N=166)**			
Non	40 (37,4)	67 (62,6)	0,32
Oui	25 (42,4)	34 (57,6)	
**Consanguinité avec le conjoint (N=164)**			
Non	46 (39,3)	71 (60,7)	0,52
Oui	18 (38,3)	29 (61,7)	
**Couverture sanitaire (N=154)**			
Non	52 (36,1)	92 (63,9)	0,49
Oui	3 (30,0)	7 (70,0)	
**Antécédent d’anomalie des grossesses antérieures (N=137)**			
Non	36 (36,7)	62 (63,3)	0,29
Oui	17 (43,6)	22 (56,4)	
**Antécédents familiaux d’HTA ou de diabète (N=160)**			
Non	36 (42,9)	48 (57,1)	0,07
Oui	23 (30,3)	53 (69,7)	
**Utilisation des soins de santé maternelle avant le projet (N=153)**			
Non	39 (35,8)	70 (64,2)	0,50
Oui	15 (34,1)	29 (65,9)	
**Anomalie perçue par la femme au cours de la grossesse actuelle (N=145)**			
Non	18 (47,4)	20 (52,6)	0,04
Oui	32 (29,9)	75 (70,1)	

## Discussion

Dans les pays en développement, la sous-utilisation des soins de santé maternelle demeure une problématique surtout lorsqu'il s'agit d'une population rurale [9]. Le Maroc est parmi les pays où l'amélioration de la santé maternelle est une priorité nationale. Les multiples programmes mis en place visent entre autre l'augmentation du recours des femmes à la consultation prénatale chez un personnel qualifié [10]. Mais malgré les actions entreprises les résultats demeurent au-dessous des attentes en particulier au rural (91,6% en urbain contre 62,7 % en rural en 2011) [11]. L'implication de la population rurale est une clé pour assurer la pérennité des résultats de tels programmes. La démarche communautaire qui est à la base de notre projet est l'un des axes stratégiques d'intervention pour promouvoir la santé maternelle recommandés par l'OMS [12, 13]. Elle est définie comme un processus de mobilisation sociale au sein d'une communauté lui permettant d'identifier ses besoins en santé ainsi que les mécanismes d'action pour répondre à ses besoins [14]. Elle permet *l'empowerment* et l'acquisition d'un certain degré d'autonomisation chez les individus, et ainsi elle est largement reconnue pour ses avantages en termes d'amélioration de la santé maternelle surtout en première ligne de soins en milieu rural. Les résultats dépendent du degré d'engagement de la population dans les actions d'interventions [15, 16]. Nous avons adopté le modèle de la recherche action pour monter notre projet puisqu'il s'adapte au contexte de notre travail [17]. Sur une population cible de femmes enceintes estimée à 500 au cours de l'année de l'étude, 283 femmes ont consulté, ce qui correspondait à un taux de participation de 56,6% pour l'incidence attendue des grossesses. Les participantes étaient de jeune âge (moyenne de 27 ans), de faible niveau d'instruction et sans couverture sanitaire. Au regard des coutumes locales marquées par le mariage précoce, faire adhérer ces jeunes femmes était un résultat positif qui témoigne d'un degré d'autonomisation engendré. Au regard de leur âge et leur situation sociale, ces femmes sont dépourvues d'un pouvoir décisionnel concernant leur santé maternelle, et les décisions reviennent en général aux conjoints et à la famille. La plupart d'entre elles étaient multipares et n'avaient pourtant aucun contact antérieur avec le système de soins avant le projet (73,4%, N = 252). Le taux de suivi de la grossesse était un indicateur indirect de l'adhésion de l'entourage familial. Toutefois cette évaluation reste subjective devant la difficulté de mesurer le lien entre l'autonomisation des femmes et les résultats en santé maternelle décrite dans la littérature [18].

Seules 26,6% des répondantes ont déjà bénéficié d'une CPN, ce qui reste très faible par rapport aux chiffres enregistrés dans d'autres régions du Maroc. Les anomalies de la grossesse auto déclarées, dont l'infection et l'anémie ont été surestimées par les femmes (55,1%). Cette proportion élevée ne correspondait pas aux cas réellement dépistés et traités durant le projet. Ce constat peut être rattaché à plusieurs facteurs dont l'analphabétisme de la population et son contact restreint avec le système de soins qui font que les femmes n'ont pas de connaissances suffisantes. Le terme infection, par exemple, est employé pour désigner toute sorte d'anomalies uro-génitales sans qu'il y ait un diagnostic préalable. Cette perception du risque par les femmes était significativement liée à l'adhésion au suivi de la grossesse. Elle pourrait implicitement exprimer la demande d'une attention particulière de la part des intervenants au cours de la consultation. En effet, dans des contextes culturels similaires et dans telles conditions de vulnérabilité socioéconomiques, il est important de prendre en considération les représentations sociales de la population dans l'analyse de la demande pour identifier le besoin réel: soins, informations, ou simple écoute afin d'établir une relation de confiance et augmenter la fréquentation des structures de soins à moyen et long terme. L'un des objectifs de notre étude était d'augmenter le pourcentage des CPN réalisées au niveau de la zone d'étude. Le centre de santé local qui dessert une population qui avoisine 20000 habitants fonctionnait avec un seul infirmier de sexe masculin au cours de la période du projet. La fréquentation des femmes enceintes de cette structure était limitée aux cas compliqués qui nécessitaient un transfert au 2^e^ ou 3^e^ niveau de soins. Ainsi, à travers la mobilisation de la communauté, on a noté une augmentation du nombre de CPN enregistrées par rapport à l'année précédente (54% versus 8% en 2013) soit une multiplication de 6 fois, sachant que le taux de naissance est stable d'une année à l'autre.

La proportion des femmes ayant bénéficié de trois consultations prénatales était de 6,7%, et celles qui ont consulté 2 fois était de 30,4%. En retranchant les participantes vues d'emblée au 3ème trimestre de leur grossesse (au nombre de 109), 39,7% était la proportion des non adhérentes perdues de vue (N = 174) et 60,3% (au nombre de 105) des participantes ont adhéré à la surveillance de leur grossesse dans le cadre du projet par la consultation au moins deux fois. Ce résultat était plus notable en présence d'une couverture sanitaire, d'un antécédent familial d'HTA ou de diabète ou d'une anomalie du déroulement de la grossesse déclarée par la femme. Mais l'étude de ces facteurs était statistiquement non significative sauf pour la perception d'anomalies (p=0,04). Lors de l'analyse de ces facteurs, il est à noter que l'inaccessibilité géographique aux structures de soins; largement étudiée dans la littérature, a représenté un critère de base pour le choix du site d'implantation de notre projet. L'éloignement du centre de santé locale évalué en durée nécessaire de déplacement était un facteur présent chez toutes les participantes.

Dans la littérature, plusieurs expériences similaires ont rapporté leurs impacts positifs surtout en milieu rural [19, 20]. L'Afrique était parmi les zones les plus concernées par ces interventions, vu qu'elle note un taux de mortalité maternelle élevé. Cependant, les stratégies, les axes d'interventions et la durée diffèrent d'une étude à l'autre. Au cambodge, Jon skinner *et al*. ont rapporté une augmentation du taux de CPN de 22% suite à l'implication de plusieurs intervenants (accoucheuses traditionnelles, sages-femmes et villageois) dans une approche participative de la communauté lors d'un projet d'une année [21]. Notre projet qui avait une durée semblable a permis de faire passer le taux de CPN de 8% à 56,6%. Il est admis que l'utilisation des soins prénatals et le respect du nombre de consultations recommandées influencent le choix de l'accouchement dans un milieu surveillé [22]. Les consultations prénatales ont été organisées en parallèle avec des séances de sensibilisation et d'éducation pour la santé maternelle, animées par des infirmières et des médecins ayant une expérience dans l'animation des classes des mères qui se sont déroulées dans la langue locale pour une meilleure interaction avec les femmes. Notre projet est un exemple de démarche d'ouverture de la faculté de médecine sur son environnement. Il avait l'avantage d'encourager la collaboration entre la société civile et la faculté à travers la responsabilisation d'étudiant en médecine sur le plan social. L'adoption d'une approche participative, et la réorientation des services étaient les principaux axes d'action, ce qui a permis une amélioration significative du nombre de consultations prénatales. La principale limite était le biais de perdues de vue, inévitable au suivi de cohorte. Ainsi que le caractère fluctuant des volontaires au cours du projet parmi les étudiants en médecine avec des fois des effectifs insuffisants au regard de la charge des activités organisées en parallèle à chaque consultation.

## Conclusion

Le présent travail est un modèle de projet à base communautaire qui a adopté une approche de promotion de la santé et de partenariat avec la population en vue d’apporter une meilleure réponse à ces attentes de santé. Dans un contexte difficile marqué par la pénurie des ressources et par l’intrication des déterminants, ce travail pourrait servir de modèle d’intervention à encourager et à répliquer dans des zones rurales similaires au niveau et en dehors du Maroc.

### Etat des connaissances actuelles sur le sujet

- La promotion de la santé maternelle est une préoccupation de santé des pays en développement;
- La participation active de la femme et de la population est une clé de réussite des programmes de promotion de la santé.

### Contribution de notre étude à la connaissance

- La démarche de mobilisation sociale et communautaire améliore le recours et l’utilisation des soins maternels;
- Les projets de promotion de la santé à base communautaire peuvent représenter une alternative pour la résolution des problèmes de santé maternelle.

## Conflits d’intérêts

Les auteurs ne déclarent aucun conflit d'intérêts.
